# Curcumin Protects against CCl_4_-Induced Liver Fibrosis in Rats by Inhibiting HIF-1α Through an ERK-Dependent Pathway

**DOI:** 10.3390/molecules191118767

**Published:** 2014-11-17

**Authors:** Yanling Zhao, Xiao Ma, Jiabo Wang, Xuan He, Yan Hu, Ping Zhang, Ruilin Wang, Ruisheng Li, Man Gong, Shengqiang Luo, Xiaohe Xiao

**Affiliations:** 1 China Military Institute of Chinese Medicine, 302 Military Hospital of China, Beijing 100039, China; 2 Pharmacy College, Chengdu University of Traditional Chinese Medicine, Chengdu 611137, China; 3 State Key Laboratory of Biotherapy, West China Hospital, Sichuan University, Chengdu 610041, China; 4 Research and Technology Service Center, 302 Military Hospital of China, Beijing 100039, China; 5 Department of Integrative Medical Center, 302 Military Hospital of China, Beijing 100039, China

**Keywords:** curcumin, carbon tetrachloride, liver fibrosis, hypoxia inducible factor

## Abstract

The ERK/HIF-1α signaling pathway is believed to play an important role in the genesis of progressive fibrosis. An increasing expression of HIF-1α and ERK accompanies CCl_4_-induced liver fibrosis in rats. Curcumin is verified to have antifibrotic effects in several kinds of liver fibrosis models. There is no specific evidence illustrating a connection between curcumin and the HIF-1α/ERK pathway in rat liver fibrosis induced by CCl_4_. In this study, liver fibrosis was induced by CCl_4_ in treated rats. The data demonstrated that curcumin was able to attenuate liver fibrosis and inhibit the proliferation of HSC. Moreover, curcumin could remarkably elevate the hepatic function by decreasing serum levels of ALT, AST and ALP, and increasing levels of ALB, TP and α-SMA, Col III mRNA expression. Meanwhile, ECM status could also be reflected by curcumin treatment. The alleviation with curcumin treatment was associated with inhibition of HIF-1α and phosphor-ERK. This study indicates that curcumin alleviates fibrosis by reducing the expression of HIF-1α partly through the ERK pathway.

## 1. Introduction

Liver fibrosis is a reversible wound-healing response characterized by the accumulation of extracellular matrix (ECM) following liver injury [1]. It progresses to cirrhosis, liver failure, and hepatocellular cancer along with fibrogenic development. Cirrhosis, the end stage of liver fibrosis, is the leading cause of liver-disease-related morbidity and mortality worldwide [2]. Various pathological factors, such as viral hepatitis, alcohol abuse, metabolic diseases, autoimmune diseases, and cholestatic liver diseases, all contribute to the development of liver fibrosis [3]. Moreover, activation of hepatic stellate cells (HSCs) has been considered as an essential event for liver fibrogenesis and leads to increased production of alpha-smooth muscle actin (a-SMA) and collagens [4]. The activated HSC causes the progressive ECM synthesis rather than degradation, resulting in accumulation of ECM, which ultimately leads to liver fibrosis [5].

The hypoxia inducible factors (HIF) are a family of evolutionarily conserved transcriptional regulators that affect a homeostatic response to low oxygen tension and have been identified as key mediators of angiogenesis, inflammation, and metabolism [6]. HIF-1, as one member of HIFs, is composed of a stable HIF-1β subunit and a labile HIF-1α subunit. Evidence-backed studies reveal that HIF-1α is able to upregulate the expression of vascular endothelial growth factor (VEGF), platelet derived growth factor (PDGF), tissue inhibitor of metalloproteinase (TIMPs) and connective tissue growth factor (CTGF) and all these factors play an important role in fibrogenesis [7,8,9,10]. Growing evidence has suggested that expression of HIF-1α is upregulated by the Ras/MAPK pawthway. For example, Sutton *et al.* found that overexpression of the ERK 1 could significantly improve the activity of HIF-1 [11]. Wang *et al.* reported that inhibition of MAPK phosphorylation enhanced HIF-1α ubiquitination and inhibited HIF-1α translocation into nucleus, which was associated with activation in HSC-T6 proliferation [10]. Therefore, inhibition of HIF-1α through ERK-dependent pathway plays an important role in HSC inhibition.

**Figure 1:**
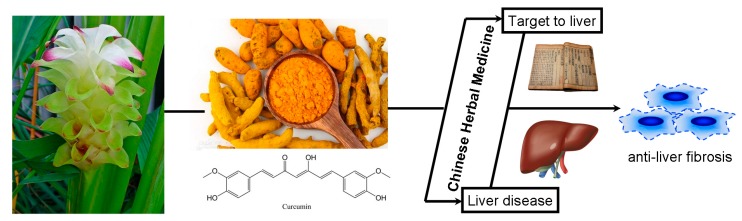
Overview of our study.

Curcumin, a polyphenol, is the main active compound derived from the perennial plant *Curcuma longa*. It has been used as an anti-oxidant and anti-inflammation remedy in Chinese herbal medicines (CHM) for hundreds of years. Recently, curcumin has been indicated as a potential treatment for liver fibrosis through mediation of various signaling pathways. It decreased the expression of proinflammatory mediators such as tumor necrosis factor alpha (TNF-α), interleukelin-6 (IL-6) and monocyte chemotactic protein 1 (MCP-1) through down regulation of high mobility group box-1 protein (HMGB1), toll-like receptor 4 (TLR4) and TLR2 expression in CCl_4_-induced rat model of fibrogenesis [12]. Curcumin could also remarkably attenuate the severity of CCl_4_-induced liver fibrosis through inhibition of TGF-β1/Smad signaling pathway and CTGF expression [13]. In addition, attenuation of sinusoidal angiogenesis and capillarization via PPARγ activation is another mechanism for liver fibrosis prevention [14,15]. Furthermore, it similarly stimulated peroxisome proliferator-activated receptor gamma (PPARγ) activity in activated HSC, which was required for curcumin to reduce cell proliferation, induce apoptosis and suppress ECM gene expression [16]. However, there is no study on the role of the ERK/HIF-1α pathway in liver fibrosis treated with curcumin. Therefore, the purpose of the present study was to investigate whether curcumin could act as anti-liver fibrosis agent through regulation of ERK/HIF-1α in liver fibrosis in rats induced by CCl_4_, a widely used chemical to induce liver injury in rodents (Figure 1).

## 2. Results and Discussion

### 2.1. Histological Examination

**Figure 2:**
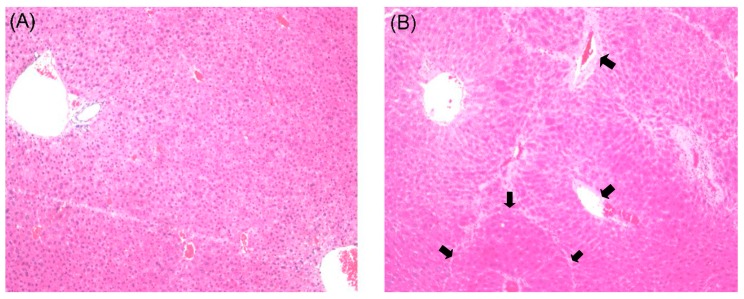

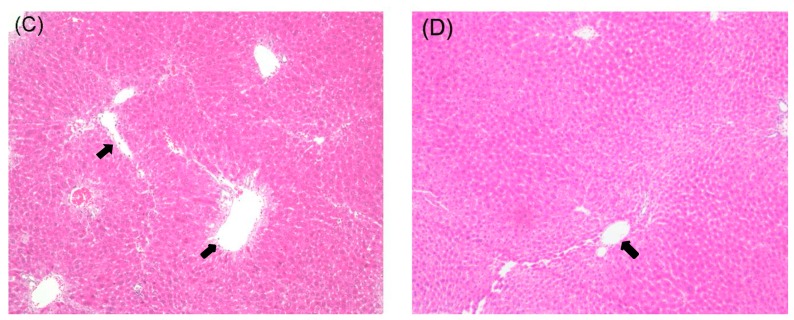
Histological examination of rat livers stained with H&E. (**A**) Normal group, without any abnormal morphological alternations; (**B**) Model group, showing marked morphological disruption; (**C**) CUR-L group, showing moderate regression of morphological changes; (**D**) CUR-H group, showing marked regression with a large amount of alleviation in the abnormal area. Original magnification 100×.

Histological evaluations provided direct assessment of the protective effects of curcumin on CCl_4_-induced liver fibrosis. The liver tissues in the rats of the normal group showed a natural structure without any abnormal morphological alternations in lobular architecture and hepatocytes (Figure 2A). A large amount of acidophilic degeneration, disorder of partial hepatic plates with stretching perivascular fibrosis could be found in the model group (Figure 2B). The groups treated with low and high doses of curcumin appeared to relieve the pathological damages in different degrees comparing with model group respectively. The CUR-L group displayed a moderately reduced severity of hyperplasia in liver tissue and hepatic plate (Figure 2C). Furthermore, the CUR-H group demonstrated a large amount of alleviation in abnormal area compared with model group and almost similar to normal group (Figure 2D).

### 2.2. Effects of Curcumin on Serum Biochemistry

Table 1 lists serum levels of ALT, AST, ALP, ALB and TP, five sensitive indicators of liver damage and hepatic function. Compared with the normal group, serum ALT, AST and ALP levels increased 2.5-, 2.8- and 1.6-fold, respectively, and serum levels of ALB and TP levels decreased 0.56- and 0.6-fold, respectively. High levels of ALT, AST and ALP induced by CCl_4_ were alleviated significantly in both high and low dose curcumin-treated rats. In addition, low levels of ALB and TP induced by CCl_4_ increased remarkably in high and low dose curcumin-treated rats. Furthermore, ALB and TP levels of high and low dose curcumin-treated rats were lower than levels of normal rats, indicating weaker regulation of ALB and TP.

**Table 1:** Effects of curcumin on serum ALT, AST, ALP, ALB, TP (mean ± SD).

Group	ALT (U/L)	AST (U/L)	ALP (U/L)	ALB (g/L)	TP (g/L)
Normal	60.06 ± 15.04	140.23 ± 19.27	270.96 ± 17.82	41.45 ± 2.84	70.90 ± 6.43
Model	158.10 ± 23.50 **	394.28 ± 37.74 **	434.42 ± 52.30 **	23.95 ± 3.05 **	42.10 ± 5.13 **
CUR-L	52.98 ± 12.36 ##	106.67 ± 19.26 ##	260.78 ± 22.99 ##	29.72 ± 3.27 **##	49.17 ± 5.49 **#
CUR-H	53.02 ± 15.20 ##	100.48 ± 17.25 ##	241.42 ± 33.11 ##	30.60 ± 3.17 **##	53.67 ± 7.68 **##

Data were expressed as mean ± SD. * *p* < 0.05, ** *p* < 0.01 compared with normal group, # *p* < 0.05, ## *p* < 0.01 compared with model group.

### 2.3. Effects of Curcumin on mRNA Expression of α-SMA and Col III

**Figure 3:**
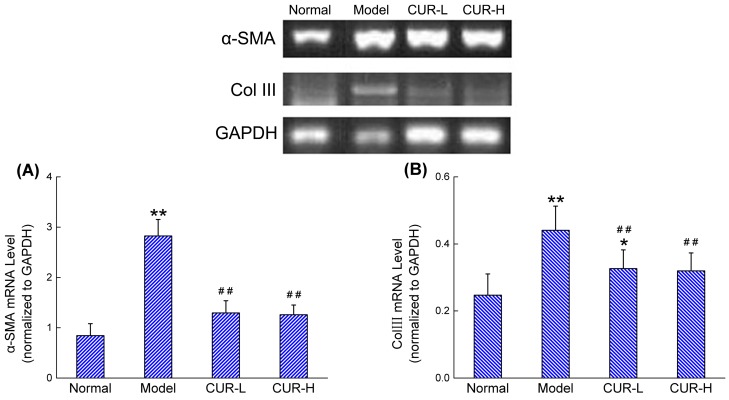
Effects of curcumin on mRNA expression of α-SMA and Col III in liver tissues. The sample tested in this experiment was abstracted from the liver tissues of rats: (**A**) α-SMA; (**B**) Col III. Data were expressed as mean ± SD. * *p* < 0.05, ** *p* < 0.01 compared with normal group, # *p* < 0.05, ## *p* < 0.01 compared with model group.

As shown in Figure 3, the mRNA level of α-SMA was significantly increased in the model group compared with the normal group. In CUR-L and CUR-H groups, the mRNA level of α-SMA was decreased compared with the model group, and was even similar to the normal group. Meanwhile, the mRNA level of Col III was also increased significantly in the model group compared with that in the normal group. Furthermore, curcumin was able to attenuate the upregulation of Col III. However, the Col III expression of low dose curcumin group was also remarkably higher than the expression of normal group, indicating a weaker effect in low dosage (Figure 3B).

### 2.4. Effects of Curcumin on Activation of HIF-1α and p-ERK

**Figure 4:**
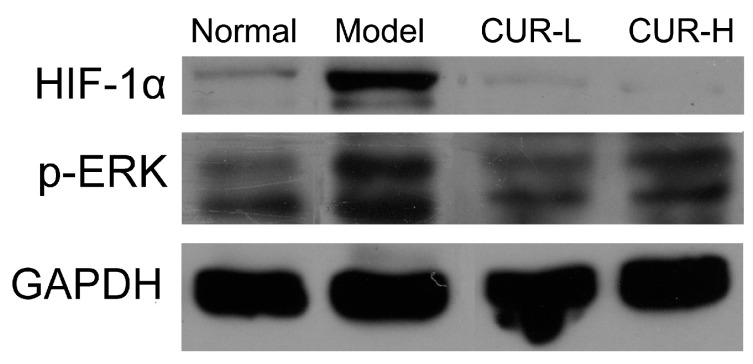

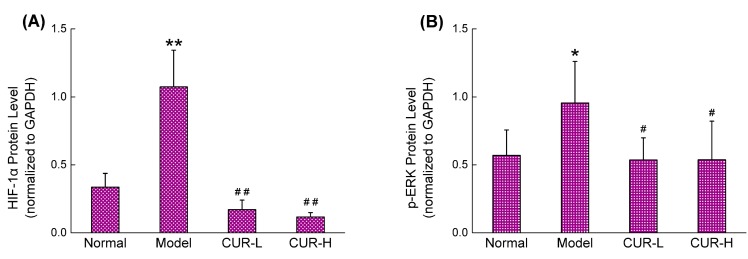
Effects of curcumin on the expression of HIF-1α and p-ERK in liver tissues. The protein used in this experiment was extracted from the liver tissues of rats: (**A**) HIF-1α; (**B**) p-ERK. Data were expressed as mean ± SD. * *p* < 0.05, ** *p* < 0.01 compared with normal group, # *p* < 0.05, ## *p* < 0.01 compared with model group.

The western-blot analysis revealed a marked 3.2- and 1.7-fold increase of HIF-1α and p-ERK in the model group compared with that in the normal group (Figure 4). The CUR-L and CUR-H treated groups, protein levels of HIF-1α both decreased significantly (Figure 4A). Protein levels of p-ERK also decreased significantly in both the CUR-L and CUR-H groups to a level comparable with the normal group (Figure 4B).

### 2.5. Cell Viability of Curcumin on HSC-T6 in Vitro

**Figure 5:**
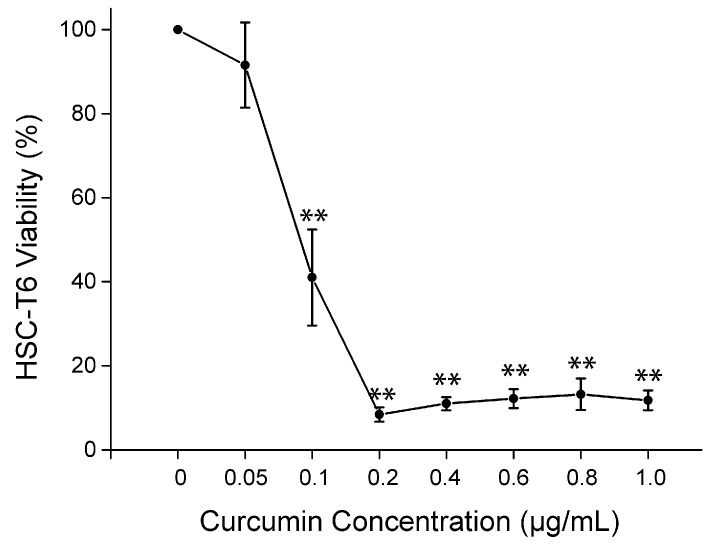
Cell viability of curcumin on HSC-T6 *in vitro*. Curcumin marked decreased the viability of HSC-T6 at the concentration of 0.1 μg/mL, and the inhibition increased from 0.05 μg/mL to 1.0 μg/mL. ** *p* < 0.01 compared with HSC-T6 treated with solvent but no curcumin.

We investigated the effect of curcumin on the HSC cell line *in vitro* because HSC plays an important role in liver fibrosis. HSC-T6, a well-characterized rat HSC cell line, recapitulates many features of the activated HSC phenotype. After treatment with curcumin, cell viability of HSC-T6 was significantly reduced from 0.1 μg/mL to 1.0 μg/mL (*p* < 0.01) (Figure 5). Moreover, the cell viability rate reduced below 14% with the concentration of 0.2 μg/mL. This indicated a potent inbihition of HSC proliferation from 0.2 μg/mL to 1.0 μg/mL.

### 2.6. Discussion

CHM has been used as complementary and alternative therapy in the prevention of liver fibrosis for centuries. Currently, a number of compounds exhibiting anti-liver fibrosis effects have been discovered in CHM, such as oxymatrine [17], matrine [18], tetrandrine [19], silybin [20], puerarin [21], quercetin [22], salvianolic acid B [23], paeoniflorin [24] and so on. Curcumin, an effective anti-liver fibrosis compound, demonstrates its efficacy through various signaling pathways. However, there is no specific report on the anti-fibrosis effects of curcumin through the ERK/HIF-1α pathway. This study sought to explore the efficacy of curcumin on this specific fibrogenesis pathway.

HSC activation is thought to represent the crucial step of fibrogenesis. In this study, the results illustrated that administration of curcumin from 400 to 1200 mg/kg remarkably attenuated the degree of fibrosis, confirming its antifibrotic effects. Significantly distorted architecture and increasing ductules were found in CCl_4_-treated rats. Meanwhile curcumin could alleviate the abnormal changes in CCl_4_-induced fibrotic liver. In the model group which rats were treated with CCl_4_-increased serum biomarkers of ALT, AST, ALP and decreased levels of ALB, TP could be found. These indicators were reversed after treated with curcumin in both high and low doses. As the most sensitive indicators in liver disease, ALT, AST are hepatocyte cytosolic enzymes while ALP is an enzyme in the liver cytoplasm outside, the increased levels of ALT, AST and ALP usually indicate cell damage. [25]. ALB and TP levels tend to be reduced in chronic liver injury as a result of the impaired ability of liver cells to synthesize proteins [26]. In our study, we observed decreased serum ALT, AST and ALP levels and increased serum ALP and TP levels in the CUR-L and CUR-H groups, indicating the liver protective effects of curcumin. The common dose used in previous reports was 100 mg/kg to 400 mg/kg. In this dose range, the antifibrotic effect was enhanced as the dose increased. However, no data on liver fibrosis were reported for high dose applications above 400 mg/kg. In this study, we found that when we increased the dose, the effect was not enhanced up to the maximum safe dose of 1200 mg/kg. Therefore, the result partially coincided with previous reports where we treated rats with 400 mg/kg. Although the effect of 1200 mg/kg curcumin was similar to that of 400 mg/kg, it gave us a more comprehensive view of the effects of high dose application.

Collagen is also a sensitive index which reflects the fibrosis level and accounts for about 50% of the total protein in fibrous liver [27]. In this study, levels of Col III in CCl_4_-treated rats increased significantly compared with normal rats. Curcumin was able to attenuate the up-regulation of Col III. Moreover, α-SMA is a specific marker of HSCs and myofibroblasts with the characteristics of acting in the identification and quantification of activated HSCs in liver fibrosis progression [28]. Expression of α-SMA gene in liver fibrosis rats markedly increased after CCl_4_ treatment for six weeks. Curcumin at 400 and 1200 mg/kg both could remarkably attenuate the level of α-SMA gene expression, indicating that curcumin was potent and specific in its anti fibrogenesis action. In addition, we also investigated the effect of curcumin on the HSC-T6 cell line *in vitro*. The results showed that the cell viability of HSC-T6 was significantly reduced from 0.1 to 1.0 μg/mL, demonstrating a remarkable inhibition of HSC proliferation when exposed to curcumin. The results found in this experiment revealed that curcumin was an effective and potent agent for liver fibrosis.

**Figure 6:**
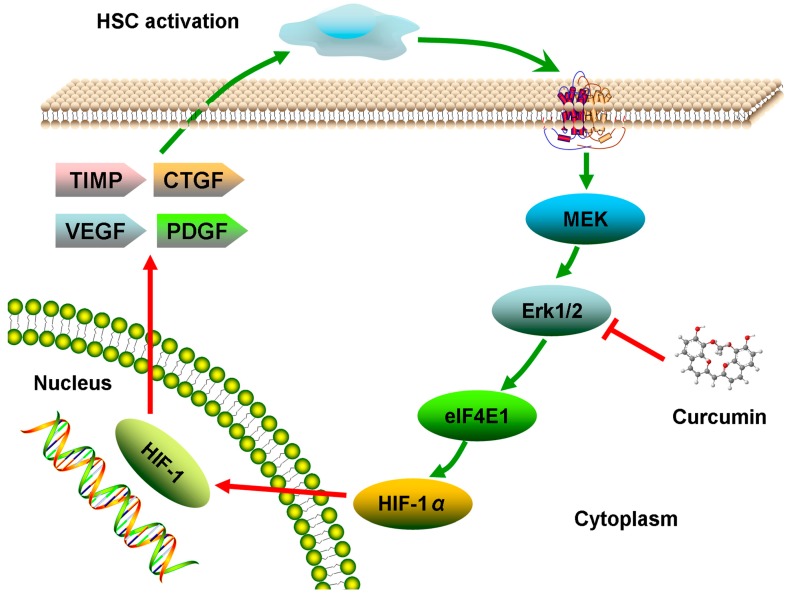
HIF-1α regulation through the ERK signaling pathway in liver fibrosis.

HIF-1, associated with inflammation and angiogenesis, plays an important role in the genesis and progress of tissue fibrosis [29]. Once the tissue is injured, the apoptosis of hepatocytes can trigger a hypoxic environment and this induces the activation of HIF-1. The active HIF-1, consisting of an alpha and a beta subunit, could translocate to the nucleus, where it binds to hypoxia-responsive element (HREs) in the promoter region of target genes. HIF-1 targets gene families that represent proinflammatory and profibrotic mediators, as well as genes involved in tumor progression [30]. Recent evidence indicates that there is a profound effect of HIF-1 which can induce TIMP-1, PAI-1, and CTGF transcription. Research found that nuclear HIF-1α protein was increasingly presented and displayed as an important regulator of profibrotic mediator production by macrophages during the development of liver fibrosis [31]. ERK, upstream of HIF-1α, and an important marker in fibrogenesis, has been reported to elevate the expression of HIF-1 and increase its activity by direct phosphorylation or indirect phosphorylation [32]. Consistently, we found elevated expression of HIF-1α and p-ERK in model group, but decreased expression of HIF-1α and p-ERK in CUR-L and CUR-H groups. Based on these pieces of evidence and our results, we believe that HIF-1α may promote liver fibrosis, at least, partly through the ERK pathway (Figure 6).

In further analysis, studies using advanced technology, such as genomics and metabonomics [33,34], should be included to investigate the underlying mechanism(s) of action of curcumin. Chinese medicines are usually multicomponent systems with a herb consisting of several active compounds. Plenty of studies demonstrate that CHM with definite efficacy for specific diseases regulate the function of multiple organs and tissues in a network manner [35,36].

## 3. Experimental Section 

### 3.1. Chemical and Reagents

Curcumin, purchased from Xi’an Haoxuan Biotechnology Limited Company (Xi’an, China), was more than 95% pure by ultra-performance liquid chromatography (UPLC) analysis. CCl_4_ was purchased from Beijing Jingxi Chemical Company (Beijing, China, batch number: 20130604). Olive oil was purchased from the Chinese medicine group, Shanghai Chemical Reagent Company (Shanghai, China, batch number: F990920). TRIzol was purchased from Invitrogen (Carlsbad, CA, USA). M-MLV transcription reagents were purchased from Takara (Otsu, Japan). All primers were synthesised by Beijing Sanboyuanzhi Biotechnology LLC (Beijing, China). Test kits for qlbumin (ALB) (batch number: 140913014), total protein (TP) (batch number: 140813013), alanine aminotransferase (batch number: 140113021), aspartate aminotransferase (batch number: 140213018), alkaline phosphatase (ALP) (batch number: 140313008) were purchased from Shenzhen Midray Biomedical Electron Corporation (Shenzhen, China). HIF-1α antibody, phosphor-p44/42 MAPK(Erk 1/2) (Thr202/Tyr204) XP^®^ rabbit mAb, GAPDH (14C10) rabbit mAb, anti-rabbit IgG HRP-linked antibody were all purchased from Cell Signaling Technology Company ( Danvers, MA, USA).

### 3.2. Animals

Forty Male Sprague-Dawley (SD) rats weighing 180–200 g were obtained from the laboratory animal center of The Military Medical Science Academy of the PLA (Permit No. SCXK-(A) 2012-0004). All animals received humane care in compliance with the Chinese Animal Protection Act, according to the National Research Council criteria. The animals were maintained under controlled conditions of temperature 20 ± 0.5 °C, humidity 55% ± 5% and with 12 h light and 12 h dark cycles. Water and food were made available to the rats *ad libitum*.

### 3.3. Treatment Regimens/Experiment Protocols

After a 5-day acclimation period, rats were randomly divided into four groups including a normal group (*n* = 10), model group (*n* = 10), and curcumin (400, 1200 mg/kg) treated groups (*n* = 10 each). One group was used as control with olive oil the same volume of other groups but without CCl_4_. The other three groups were intraperitoneally injected CCl_4_ at a dose of 500 μL/100 g (mixed 1:1 with olive oil) body weight for the first time and following at a dose of 300 μL/100 g (mixed 3:7 with olive oil) in order to induce liver fibrosis. The injections were given twice a week over a period of six weeks based on our preliminary tests with the results showing that the liver fibrosis model conformed to the model in the published articles [13,37]. Curcumin, dissolved in normal saline at the selected concentrations, was administered orally by gavage once a day over the 6 weeks at the dose of 400 (low dose), 1,200 (high dose) mg/kg, respectively. Normal saline was given as control. At the end of the experiment, all rats were sacrificed. Samples of liver tissue and serum were collected for further analysis.

### 3.4. Measurement of Serum Biomarkers ALT, AST, ALP, ALB and TP 

Blood (10 mL) was collected at rats sacrifice and centrifuged 3000 g for ten min. The serum biomarkers, including ALT, AST, ALP, ALB and TP, were determined using clinical test kits according to the corresponding manufacturer’s instructions.

### 3.5. Histological Assessment

The left lobe of the liver of each rat were fixed in 4% buffered paraformaldehyde, dehydrated with different graded alcohol series, embedded in paraffin and and cut into 5 μm sections. Sections were stained with hematoxylin and eosin (H&E). The stained sections were examined under a Nikon microscope and analyzed by image Pro-Plus 7200 software.

### 3.6. RT-PCR Analysis for Collagen III and α-SMA

The effects of curcumin on collagen III (Col III), α-SMA mRNA expression of liver tissue from fibrotic rat models were determined by RT-PCR. Total RNA was extracted from liver tissues of each group following the manufacturer’s protocols using Trizol reagent. RNA concentration was determined by optical density measurement at 260 nm on a spectrophotometer. RNA (2 μg) was reverse-transcribed using a PrimeScript^TM^ RT reagent kit, and 2 μL cDNA was used for the PCR reaction. The PCR products were determined by using 1.5% agarose gel electrophoresis and ethidium bromide (EB) staining. The gels images were analyzed using the Quantity One software. Primers used in our paper are listed in Table 2.

**Table 2:** Primers sequences for RT-PCR.

Gene	Sense Primer (5'–3')	Anti-Sense Primer (5'–3')	Product Size (bp)
GAPDH	ACAGCAACAGGGTGGTGGAC	TTTGAGGGTGCAGCGAACTT	150
α-SMA	CTGCTTCTCTTCTTCCCT	GCCAGCTTCGTCATACTCC	410
ColIII	GTCCACAGCCTTCTACAC	CATCAAAGCCTCTGTGTC	540

### 3.7. Western Blot Analysis for ERK and HIF-1α

Liver sections were homogenated in lysis buffer (20 mM HEPES, 2 mM MgCl_2_, 1 mM EDTA, 1 mM DTT, 0.1% SDS, 1 mM PMSF, pH 7.4) on ice. The supernatants were harvested by centrifugation at 12,000 g at 4 °C for 10 min. Protein concentrations were determined by a Bradford assay. Protein (20 μg) was separated on a 12% SDS-PAGE gel and transferred to PVDF membranes. Membranes were blocked for 1 h at room temperature with 5% nonfat milk in TBS-Tween 20 (0.1% TBST) and incubated with corresponding primary antibodies including rabbit mAb of p-ERK1/2, HIF-1α and GAPDH. For protein quantification, bands were scanned and quantified with GAPDH as an internal control. The membranes were incubated with ECL reagent for 2–10 min and exposed to X-ray film.

### 3.8. Effect of Curcumin on HSC-T6 in Vitro

The HSC-T6 cell line sample was kindly provided by Professor FeiYe (Institute of Materia Medica, Chinese Academy of Medical Sciences). HSC-T6 cells were cultured in DMEM medium supplemented with 10% FBS and then seeded in a 96-well plate with 200 μL (5 × 10^4^ cells/mL) per well. After overnight incubation, cells were exposed to curcumin dissolved in 0.1% dimethyl sulfoxide (DMSO) at different concentrations (1.0, 0.8, 0.6, 0.4, 0.2, 0.1, 0.05, 0 μg/mL) for 24 h. Untreated cells were used as controls. Cell viability was evaluated using a 3-(4,5-dimethythiazol-2-yl)-2,5-diphenyltetrazolium bromide (MTT) assay. Briefly, MTT solution (20 μL, 5 mg/mL, diluted in PBS) was added to each well and the plate was incubated at 37 °C in a 5% CO_2_ atmosphere for 4 h. Then the supernatants were removed and 100 μL/well DMSO was added to dissolve formazan crystals. The absorbance at 570 nm was read using EL800 (BIO-TEC Instruments Inc, Winooski, VT, USA) microplate reader.

### 3.9. Statistical Analysis

Data were expressed as means ± standard deviation (SD). One-way analysis of variance (ANOVA) was used to compare the difference between groups. *p* values less than 0.05 or 0.01 were considered statistically significant or highly significant. All statistical analysis was performed using SPSS software, version 13.0 (SPSS Inc, Chicago, IL, USA).

## 4. Conclusions

In summary, we have observed anti-liver fibrosis effects of curcumin in rats and cells with the results showing that curcumin is beneficial for rats with liver fibrosis through inhibition of cell proliferation and suppression of expression of Col III and α-SMA genes. Furthermore, the mechanism of this function is related to decreasing HIF-1α and ERK1/2 expression. Our results provide evidence that curcumin could protect against CCl_4_-induced liver fibrosis by inhibiting HIF-1α through the ERK-dependent pathway, indicating that HIF-1α might be a potential target and curcumin might be an attractive agent for treatment of liver fibrosis.

## Abbreviations

α-SMA: alpha-smooth muscle actin
CUR: curcumin
CHM: Chinese herbal medicines
Col III: collagen III
DMSO: dimethyl sulfoxide
EB: ethidium bromide
ECM: extracellular matrix
HIF: hypoxia inducible factor
HSC: hepatic stellate cell
